# Predicting the Susceptibility of Meningococcal Serogroup B Isolates to Bactericidal Antibodies Elicited by Bivalent rLP2086, a Novel Prophylactic Vaccine

**DOI:** 10.1128/mBio.00036-18

**Published:** 2018-03-13

**Authors:** Lisa K. McNeil, Robert G. K. Donald, Alexey Gribenko, Roger French, Nathaniel Lambert, Shannon L. Harris, Thomas R. Jones, Sheng Li, Gary Zlotnick, Ulrich Vogel, Heike Claus, Raquel Abad, Julio A. Vazquez, Ray Borrow, Jamie Findlow, Muhamed-Kheir Taha, Ala-Eddine Deghmane, Dominique A. Caugant, Paula Kriz, Martin Musilek, Xin Wang, Jeni Vuong, Leonard W. Mayer, Michael W. Pride, Kathrin U. Jansen, Annaliesa S. Anderson

**Affiliations:** a Pfizer Vaccine Research and Development, Pearl River, New York, USA; b Department of Medicine, University of California, San Diego, California, USA; c Institute for Hygiene and Microbiology, University of Würzburg, Würzburg, Germany; d Reference and Research Laboratory for Vaccine Preventable Bacterial Diseases, Institute of Health Carlos III, Majadahonda, Spain; e Public Health England, Manchester Royal Infirmary, Manchester, United Kingdom; f Institut Pasteur, Invasive Bacterial Infections Unit, Paris, France; g Department of Bacteriology and Immunology, Norwegian Institute of Public Health, Oslo, Norway; h National Institute of Public Health, Prague, Czech Republic; i Meningitis and Vaccine Preventable Diseases Branch, Centers for Disease Control and Prevention, Atlanta, Georgia, USA; University of Rochester

**Keywords:** meningococcal antigen surface expression (MEASURE) assay, *Neisseria meningitidis* serogroup B, factor H binding protein, flow cytometry, vaccine

## Abstract

Bivalent rLP2086 (Trumenba), a vaccine for prevention of *Neisseria meningitidis* serogroup B (NmB) disease, was licensed for use in adolescents and young adults after it was demonstrated that it elicits antibodies that initiate complement-mediated killing of invasive NmB isolates in a serum bactericidal assay with human complement (hSBA). The vaccine consists of two factor H binding proteins (fHBPs) representing divergent subfamilies to ensure broad coverage. Although it is the surrogate of efficacy, an hSBA is not suitable for testing large numbers of strains in local laboratories. Previously, an association between the *in vitro* fHBP surface expression level and the susceptibility of NmB isolates to killing was observed. Therefore, a flow cytometric meningococcal antigen surface expression (MEASURE) assay was developed and validated by using an antibody that binds to all fHBP variants from both fHBP subfamilies and accurately quantitates the level of fHBP expressed on the cell surface of NmB isolates with mean fluorescence intensity as the readout. Two collections of invasive NmB isolates (*n* = 1,814, *n* = 109) were evaluated in the assay, with the smaller set also tested in hSBAs using individual and pooled human serum samples from young adults vaccinated with bivalent rLP2086. From these data, an analysis based on fHBP variant prevalence in the larger 1,814-isolate set showed that >91% of all meningococcal serogroup B isolates expressed sufficient levels of fHBP to be susceptible to bactericidal killing by vaccine-induced antibodies.

## IMPORTANCE

Bivalent rLP2086 (Trumenba) vaccine, composed of two factor H binding proteins (fHBPs), was recently licensed for the prevention of *N. meningitidis* serogroup B (NmB) disease in individuals 10 to 25 years old in the United States. This study evaluated a large collection of NmB isolates from the United States and Europe by using a flow cytometric MEASURE assay to quantitate the surface expression of the vaccine antigen fHBP. We find that expression levels and the proportion of strains above the level associated with susceptibility in an hSBA are generally consistent across these geographic regions. Thus, the assay can be used to predict which NmB isolates are susceptible to killing in the hSBA and therefore is able to demonstrate an fHBP vaccine-induced bactericidal response. This work significantly advances our understanding of the potential for bivalent rLP2086 to provide broad coverage against diverse invasive-disease-causing NmB isolates.

## INTRODUCTION

*Neisseria meningitidis* can cause devastating invasive disease that often progresses very rapidly and is therefore difficult to diagnose and treat (1). The burden of disease is highest in children <1 year old, followed by a second peak during adolescence (2). There are 12 known serogroups of *N. meningitidis* based on different capsular polysaccharide structures, of which 6 (A, B, C, W, Y, and X) are most commonly associated with significant clinical disease (3, 4). Currently, polysaccharide conjugate vaccines (serogroups A, C, W, and Y) and outer membrane protein antigen vaccines (serogroup B) are commercially available. Polysaccharide vaccines for disease due to *N. meningitidis* serogroup B (NmB) could not be developed because of its similarity to a human neural antigen (5–7).

The search for an NmB vaccine led to the discovery of the outer membrane lipidated protein factor H binding protein (fHBP) as a vaccine candidate (8, 9). Binding of human factor H, a negative regulator of the alternative complement pathway, to fHBP helps the organism evade host innate immunity (10). fHBP (also known as LP2086) is a 28-kDa lipoprotein located in the outer membrane of NmB isolates, as well as isolates from other serogroups (9). The gene for fHBP is present in most of the meningococcal isolates examined and is an important virulence factor for survival of the bacteria *in vivo* (11–14). fHBP can be classified by amino acid sequence into two subfamilies, A and B. While the sequence similarity within each subfamily is high (>83%), the sequence similarity between the two subfamilies is only 60 to 75% (13). Thus, an effective vaccine that is based only upon fHBP must contain a lipoprotein antigen from each subfamily to be broadly protective (15). Recently, two fHBP-based vaccines, Trumenba (bivalent rLP2086) and Bexsero (4CMenB), the latter of which contains additional antigenic components, were licensed in the United States for use in 10- to 25-year-olds for the prevention of invasive NmB disease (16). Licensure was achieved after it was demonstrated that the vaccines generated antibodies that could kill representative disease-causing NmB isolates in a serum bactericidal assay with human complement (hSBA) (17–20).

The hSBA, used to measure individual vaccination response rates, also provides an indication of protection at the level of the population targeted to receive the vaccine. The meningococcal polysaccharide conjugate vaccines for *N. meningitidis* A, C, Y, and W were licensed on the basis of the SBA as an immunologic surrogate of efficacy using corresponding capsule-expressing isolates (21). However, the challenge for a noncapsular antigen vaccine is in estimating the breadth of immune protection across the sequence heterogeneity and surface expression variation present among meningococcal isolates. It is necessary to demonstrate that an hSBA response can be elicited against a large proportion of the NmB isolates in both fHBP subfamilies, regardless of sequence variation or antigen expression levels. The hSBA is not practical for testing large numbers of NmB isolates because of the serum volume required, as well as the considerable challenges in identifying appropriate human complement sources for each isolate. The pivotal immunogenicity assessment in clinical studies for the United States licensure of rLP2086 included five coprimary immunogenicity endpoints to demonstrate that the vaccine induces protective responses against four strains representing the diversity of fHBP and common circulating strains. These four isolates were selected in an unbiased fashion from a large collection of NmB isolates (i.e., a systematically assembled collection of invasive-disease-causing NmB isolates obtained from the United States, the United Kingdom, France, Norway, the Czech Republic, Spain, and Germany [*n* = 1,814]) and adjusted on the basis of fHBP variant prevalence (11, 13, 22). An earlier study demonstrated that *in vitro* fHBP surface expression could predict susceptibility to killing in an hSBA (15). To assess isolate diversity and help select representative isolates to demonstrate that the vaccine provides breadth of coverage in hSBAs in clinical studies (23), it was important to establish a reliable method to enumerate *in vitro* surface expression of fHBP on NmB isolates. Therefore, we developed and validated the meningococcal antigen surface expression (MEASURE) assay that measures the surface expression of fHBP variants on intact *N. meningitidis* isolates. The sequence diversity of fHBP made it important to identify a universal antibody for the MEASURE assay that was cross-reactive to all NmB isolates and could accurately quantify the amount of surface-expressed fHBP on diverse meningococcal isolates. With such an antibody identified and characterized, we sought to assess all of the isolates in the NmB invasive-isolate set in the MEASURE assay to characterize their surface expression and to investigate whether expression levels could predict their potential susceptibility to killing in the hSBA. A subset of isolates (*n* = 109) was further tested in an hSBA using human bivalent rLP2086 immune serum. A weighted analysis of these hSBA and fHBP expression data was conducted by using the entire NmB invasive-isolate set (*n* = 1,814) to evaluate the probability that an isolate could be killed in the hSBA.

## RESULTS

### Specificity, reactivity, and affinity of the MN86-994-11-1 MAb for fHBP.

**FIG 1:**
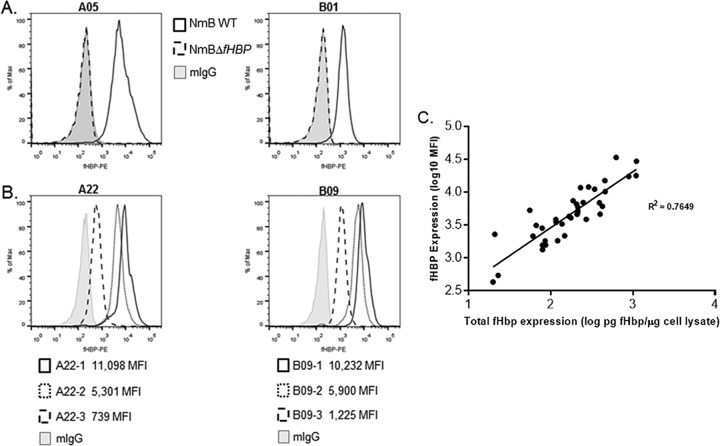
MAb MN86-994-11-1 is specific for fHBP and correlates with total cellular fHBP expression. (A) Antibody specificity was confirmed by obtaining positive MN86-994-11 detection with NmB WT isolates (black line) and no detection on corresponding fHBP NmBΔ*fHBP* isolates (dashed line), similar to the mouse IgG negative control (gray histogram). (B) Three different isolates expressing A22 fHBP variants and three different isolates expressing B09 fHBP variants were used to measure fHBP surface expression in a flow cytometry assay with MN86-994-11-1. (C) Correlation of surface expression of fHBP (MFI, log_10_) as assessed in the MEASURE assay with MN86-994-11-1 antibody versus total cellular fHBP as quantitated in whole-cell lysates by fluorescent Western immunoblotting with polyclonal bivalent fHBP A and B rabbit sera. Thirty-eight isolates expressing the following nine different fHBP variants (number of isolates of each variant tested) were tested: A05 (4), A12 (1), A22 (7), A29 (3), B03 (4), B09 (3), B16 (3), B24 (8), and B44 (5). Log-transformed data with *R*^2^ values are shown. Linear regression analysis was used to estimate levels of fHBP equivalent to respective MFIs.

The discovery of broadly cross-reacting monoclonal antibody (MAb) MN86-994-11 was a fortuitous outcome of hybridoma screening aimed at identifying fHBP antigen-specific antibodies. The antibody was not bactericidal in an hSBA and therefore has no therapeutic potential (data not shown). However, flow cytometry screening identified parent MN86-994 as uniquely cross-reactive against 42 diverse NmB isolates (20 from subfamily A and 22 from subfamily B). After subsequent cloning, the binding specificity of MAb MN86-994-11-1 for fHBP on intact bacteria in the presence of other surface components was determined by using invasive NmB isolates expressing vaccine antigens fHBP-A05 and fHBP-B01 (NmB wild type [WT]) and derived laboratory-generated *fHBP* null mutants (NmBΔ*fHBP*) (Fig. 1A). This analysis confirmed that MN86-994-11-1 is highly specific for fHBP as it bound only to NmB WT isolates and not to NmBΔ*fHBP* isolates. The use of MAb MN86-994-11-1 in the flow cytometry assay provided the means to detect fHBP on the surface of NmB bacteria across a range of expression levels, from very high to low (mean fluorescence intensity [MFI] of 11,098 to 739) for both subfamily A and B strains (Fig. 1B). To further investigate the relationship between the total bacterial cell expression of fHBP and the amount of fHBP localized on the bacterial surface, 38 isolates expressing one of nine different fHBP variants (including the eight most prevalent variants in the NmB invasive-isolate set) from both subfamilies A and B were tested by the MEASURE assay and fluorescent Western immunoblotting of cell lysates using polyclonal bivalent A and B rabbit serum samples. Surface expression of fHBP by NmB isolates expressing variants from either subfamily correlated well (*R*^2^ = 0.76) with the total fHBP expression determined by quantitative Western immunoblot analysis (Fig. 1C). An MFI of 1,000 was equivalent to approximately 30 pg of fHBP/μg of total cell protein. The quantitative Western immunoblot assay signal was linear, as demonstrated by dilutions of the cell lysate (data not shown).

In general, higher fHBP expression levels were observed for subfamily B isolates than for subfamily A isolates. To determine whether the higher expression of fHBP observed on subfamily B isolates could be due to a greater affinity of the MN86-994-11-1 antibody for subfamily B fHBP variants than for subfamily A variants, kinetic experiments were conducted to assess the affinity of MAb MN86-994-11-1 for eight different recombinant fHBP variants, four from subfamily A and four from subfamily B (Table 1). Collectively, the fHBP variants representing each subfamily were chosen for the analysis to represent all six of the major fHBP subgroups (four for A and two for B) (13, 24). The MAb exhibited high-affinity binding with subnanomolar *K*_*D*_ values and similar association and dissociation rates determined for all eight fHBP variants tested, regardless of whether they were from subfamily A or B. These data indicate that differential affinity of the MAb for subfamily A and B variants is not a factor in the generally higher surface expression levels of subfamily B fHBP-expressing isolates observed.

**TABLE 1:** MAb MN86-994-11 binds with high affinity (subnanomolar *K*_*D*_) to both subfamily A and B fHBP variants^a^

fHBP variant (% identity)	Value (error)		
	*K*_*D*_ (M)	*k*_on_ (1/Ms)	*k*_off_ (1/s)
A05 (100)	2.29E-11 (<1E-12)	3.02E+5 (2.77E+2)	6.92E-6 (6.52E-8)
A12 (85)	1.73E-11 (<1E-12)	4.09E+5 (1.27E+3)	7.06E-6 (1.94E-7)
A22 (89)	8.64E-11 (<1E-12)	2.07E+5 (2.22E+2)	1.79E-5 (9.02E-8)
A29 (93)	7.36E-11 (<1E-12)	2.22E+5 (3.51E+2)	1.63E-6 (1.37E-7)
B01 (100)	5.73E-11 (<1E-12)	3.12E+5 (2.28E+2)	1.79E-5 (6.77E-8)
B09 (88)	1.25E-11 (<1E-12)	2.30E+5 (2.86E+2)	2.88E-6 (1.08E-7)
B24 (87)	2.58E-11 (<1E-12)	2.57E+5 (3.30E+2)	6.62E-6 (8.89E-8)
B44 (91)	1.50E-10 (<1E-12)	8.28E+5 (4.76E+3)	1.24E-4 (5.95E-7)

^a^ The affinity of MN86-994-11-1 for fHBP was determined with the Octet RED96 system. Four nonlipidated recombinant subfamily A variants (A05, A12, A22, and A29) and four nonlipidated subfamily B variants (B01, B09, B24, and B44) were tested. The percent amino acid sequence identities of fHBP variants to vaccine antigens A05 and B01 are indicated.

### Binding epitope of the MN86-994-11-1 MAb.

**FIG 2:**
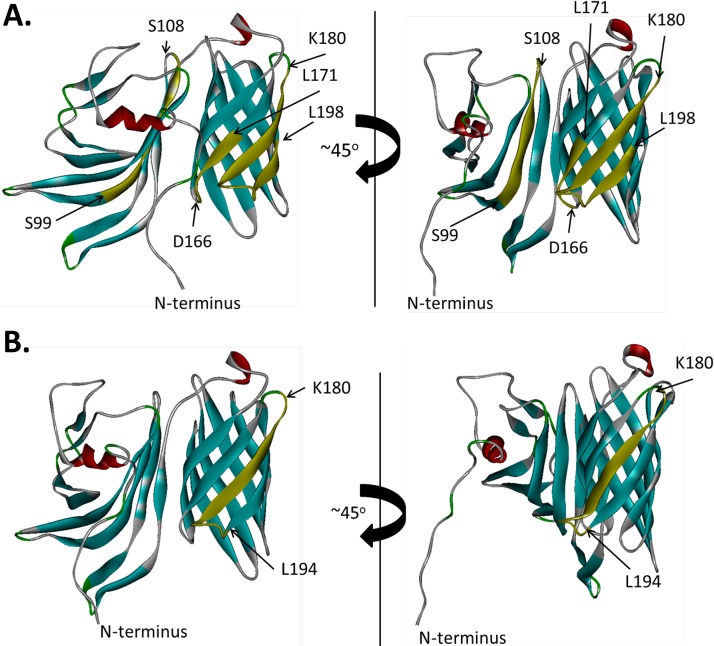
Mapping of the MN86-994-11 binding epitope on fHBP (rP2086) structural models. A schematic depiction of the binding residues of MN86-994-11-1 on fHBP is shown. A, rP2086-A05; B, rP2086-B01. Positions of the segments showing a decrease in deuterium accumulation in the presence of antibody MN86-994-11 are shown in yellow with the first and last residues of each segment highlighted.

**FIG 3:**
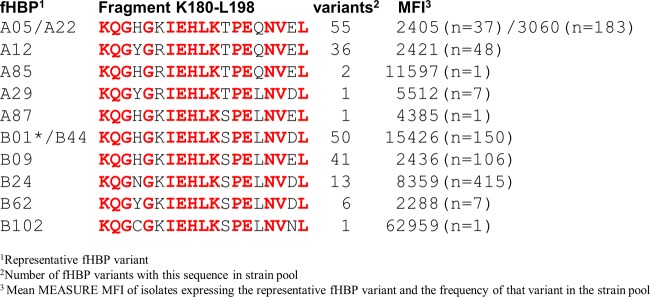
The MAb MN86-994-11-1 binding epitope is conserved among subfamily A and B fHBP variants present in the strain pool (*n* = 1,814). Peptide sequences of the MN86-994-11-1 K180-to-L198 binding site in subfamily A and B antigens defined by deuterium exchange mapping (Fig. 2) are compared. The number of variants represented by each sequence is indicated along with the mean MFI for each variant example and its corresponding frequency in the strain pool. An fHBP-B01 isolate (asterisk) is absent from the collection, but the epitope (shared with fHBP-B44) is included to permit comparisons with other fHBP variants for MAb MN86-994-11 binding affinity (see Table 1).

The binding epitopes of MAb MN86-994-11-1 on representative fHBP subfamily A and B antigens rP2086-A05 and rP2086-B01 were identified by hydrogen-deuterium exchange mass spectrometry (HDX-MS). Residues showing reduced deuterium exchange in the presence of antibody were mapped onto the three-dimensional structures of the proteins to visualize the epitopes (Fig. 2). An epitope common to both antigens was located between residues 180 and 194, which corresponds to β-strand 11 of the C-terminal β-barrel domain and the loop connecting β-strands 11 and 12. For rP2086-A05, the binding site extends through residue 198 of β-strand 12. The difference could be due to the slightly different proteolytic digest maps of the two proteins. Therefore, it is reasonable to suggest that since the binding interfaces for both antigens are similar, and given the high specificity and affinity of the antigen-antibody interactions, β-strand 12 may also be part of the MAb MN86-994-11-1 epitope for rP2086-B01. We propose that the primary binding site on both proteins is most likely a discontinuous C-terminal domain epitope spanning residues 180 to 198 on β-strands 11 and 12. The amino acid sequence conservation of this 15-amino-acid binding fragment among all of the fHBP variants present in the 1,814-strain collection was 74% identity and 89% similarity. Flow cytometry showed that isolates representing all of these sequence variants were readily detected by MAb MN86-994-11 binding to fHBP on the bacterial surface (Fig. 3).

For rP2086-A05, additional sequences beyond the common epitope also showed reduced deuterium exchange, including C-terminal residues 166 to 171 of domain β-strand 10 (immediately adjacent to the epitope) and N-terminal domain residues 99 to 108. We hypothesize that this broader binding footprint might be a consequence of greater flexibility of the rP2086-A05 tertiary structure compared with rP2086-B01, particularly with regard to interactions between the N- and C-terminal domains. Consistent with this notion, differential scanning calorimetry experiments show that the N-terminal domain of fHBP-A05 unfolds at a substantially lower temperature than that of fHBP-B01 (30.0°C compared to 55.7°C) (see Fig. S2 in the supplemental material). In addition, residues 97 to 113 of the fHBP-B01 N-terminal domain showed no deuterium exchange at all in the absence of antibody compared with the homologous fHBP-A05 segment, which appears to exchange deuterium freely and make contact with the antibody (Fig. S3). These studies support the conclusion that the MN86-994-11-1 primary binding site common to both subfamilies is a conserved conformational epitope in the C-terminal domain of fHBP.

10.1128/mBio.00036-18.1 FIG S1  Gating strategy used for the MEASURE assay. Representative gating strategy of an *N. meningitidis* sample stained with either MN86-994-11-1 or mouse IgG (negative control). (A) Forward scatter versus side scatter dot plot on a log scale with a liberal gate set around the bacterial population. (B) Histogram overlay of MN86-994-11 (aqua) and mouse IgG (red). Download FIG S1, DOCX file, 0.1 MB. Copyright © 2018 McNeil et al. 2018 McNeil et al. This content is distributed under the terms of the Creative Commons Attribution 4.0 International license.

10.1128/mBio.00036-18.2 FIG S2  Temperature-induced unfolding of fHBP monitored by differential scanning reveals that the N-terminal domain of fHBP-A05 denatures at a substantially lower temperature than fHBP-B01. All experiments were done on a VP-DSC differential scanning microcalorimeter in 1× PBS, pH 7.4, at a protein concentration of 1.3 to 1.4 mg/ml. Data were corrected for the instrumental baseline and normalized by protein concentration with Origin 6.0 software provided by the microcalorimeter manufacturer. Melting temperatures (*T*_*m*_s) were defined as maxima of the individual unfolding transitions. Download FIG S2, DOCX file, 0.03 MB. Copyright © 2018 McNeil et al. 2018 McNeil et al. This content is distributed under the terms of the Creative Commons Attribution 4.0 International license.

10.1128/mBio.00036-18.3 FIG S3  An N-terminal domain peptide of fHBP-B01 is less susceptible to deuterium exchange because of greater intrinsic stability than the homologous peptide from fHBP-A05. It is impossible to say, therefore, whether or not this structural segment is involved in MAb MN86-994-11 binding to fHBP-B01. Representative deuterium uptake plots for the proteolytic peptides derived from the N-terminal domains of fHBPs are shown. On the *y* axis is the number of protons exchanged for deuterium at each time point, and on the *x* axis is the incubation time. Blue symbols and lines represent deuterium uptake by the isolated proteins, and red lines represent deuterium uptake by the proteins in the presence of MAb MN86-994-11. Peptides are identified by the positions of the first and last residues of the protein amino acid sequence (i.e., peptides 98 to 112 cover residues 98 to 112). Download FIG S3, DOCX file, 0.2 MB. Copyright © 2018 McNeil et al. 2018 McNeil et al. This content is distributed under the terms of the Creative Commons Attribution 4.0 International license.

HDX-MS identification of the MN86-994-11 epitope was confirmed through the design of a series of mutants with amino acid substitutions within the predicted binding site. A total of 11 residues that can form hydrogen bonds or salt bridges can be found between residues 180 and 198. According to the published nuclear magnetic resonance structure (25), only residues 184 to 198 would form a continuous surface. Side chains of two residues within that stretch (H188 and E193) point away from the putative binding interface, which leaves residues K185, D187, K190, S191, N195, and D197 as potentially involved in MN86-994-11 binding. These six residues were mutated to alanine, and the ability of the mutant proteins obtained to interact with the antibody was assessed by isothermal titration calorimetry (ITC). The results are shown in Table 2. Alanine substitutions at residues K190, S191, and N195 resulted in significant (>10-fold) loss of fHBP-B01 binding affinity for MN86-994-11, indicating that these residues are directly involved in the interaction. It should be noted that the decrease in binding affinity is not due to large-scale structural disruption. All six proteins maintain well-folded secondary and tertiary structures, as evidenced by the well-defined far- and near-UV circular dichroism (CD) spectra, respectively (Fig. S4). Furthermore, the CD spectra of the mutant proteins are very similar to those of the corresponding wild-type protein, indicating that the mutations had little to no effect on protein structure. Taken together, the ITC data obtained confirm that HDX-MS identification of the MN86-994-11 epitope was correct; the antibody binds primarily to the tip of the loop connecting β-strands β3 and β4 of the C-terminal β-barrel domain of the protein.

10.1128/mBio.00036-18.4 FIG S4  CD spectra of rP2086-B01 variants. Panels: A, far-UV CD spectra (secondary structural information); B, near-UV CD spectra (tertiary structural information). Spectra of individual variants are shown in different colors, as indicated. Download FIG S4, DOCX file, 0.1 MB. Copyright © 2018 McNeil et al. 2018 McNeil et al. This content is distributed under the terms of the Creative Commons Attribution 4.0 International license.

**TABLE 2:** Thermodynamic parameters of the interaction of MN86-994-11 with rP2086-B01 epitope mutants^a^

Mutation	*K*_*a*_ (M^−1^)	*K*_*d*_ (nM)	Δ*H* (kcal/mol)	Δ*S* ([cal/mol] deg^−1^)
None (wild type)	(1.8 ± 0.2) × 10^8^	6	−11.9 ± 0.0	−2.2
K185A	(0.9 ± 0.1) × 10^8^	11	−10.8 ± 0.1	0.3
E187A	(1.2 ± 0.2) × 10^8^	8	−10.7 ± 0.1	1.0
K190A	(5.9 ± 0.4) × 10^5^	1,700	−9.3 ± 0.2	−4.9
S191A	(1.1 ± 0.2) × 10^7^	89	−8.6 ± 0.1	3.5
N195A	(4.7 ± 0.4) × 10^5^	2,100	−8.9 ± 0.3	−3.8
D197A	(6.9 ± 1.5) × 10^7^	14	−12.4 ± 0.1	−5.9

^a^ Alanine substitution mutations were introduced into conserved amino acid residues located within the epitope predicted by HDX-MS mapping (Fig. 2 and 3). Residues were targeted on the basis of their orientation and potential for forming hydrogen bonds or salt bridges with the antibody. The parameters were derived from the ITC titrations of the antibody with the wild-type and mutant proteins, as described in the text. Data are from single experiments with each protein. The errors shown are errors of fit.

### MEASURE assay validation.

The MEASURE assay assesses the surface expression of fHBP on intact fixed bacteria by flow cytometry using broadly cross-reactive MAb MN86-994-11-1 at a concentration that demonstrated saturation across variants from subfamilies A and B (6.7 μg/ml; Table S2). As shown above, this MAb binds to a conserved epitope of fHBP variants from both subfamilies and therefore can be used to quantify the level of fHBP surface expression on *N. meningitidis*. Whole intact bacteria are used so that the assay measures the antigen in its native configuration on the cell surface as it is displayed to the immune system. NmB isolates were grown under the same culture conditions used to prepare isolates for the hSBA. fHBP surface expression was subsequently evaluated by flow cytometry by a three-step staining method to amplify the fluorescence signal and increase the dynamic range of the assay. The MFIs determined in the assay were then used to compare and rank order isolates by fHBP surface expression level.

10.1128/mBio.00036-18.7 TABLE S1  NmB invasive-isolate set. The regional ABCs sites cover ~13% of the U.S. population, and all of the isolates are included in the pool. European collections survey the entire countries, and every eighth isolate (12.5%) from the available collection of each country is included in the pool. †, England, Wales, and Northern Ireland. Download TABLE S1, DOCX file, 0.02 MB. Copyright © 2018 McNeil et al. 2018 McNeil et al. This content is distributed under the terms of the Creative Commons Attribution 4.0 International license.

10.1128/mBio.00036-18.8 TABLE S2  Titration of MAb MN86-994-11-1 against subfamily A and B variants. The shaded row represents the fHBP MFI determined at the concentration of MN86-994-11-1 used in the MEASURE assay. Download TABLE S2, DOCX file, 0.02 MB. Copyright © 2018 McNeil et al. 2018 McNeil et al. This content is distributed under the terms of the Creative Commons Attribution 4.0 International license.

The MEASURE assay was validated to establish its suitability for reliably determining fHBP surface expression. Specificity and precision (repeatability and intermediate precision) were examined. Repeatability measures assay variability within an assay run, whereas intermediate precision measures assay variability over time within a laboratory while taking into account relevant sources of variation in the operating conditions (e.g., different analysts, assay days, etc.). A panel of seven meningococcal isolates was tested in the MEASURE assay to confirm repeatability and intermediate precision. These isolates cover a wide range of fHBP surface expression levels (from low to high), encompass fHBP variants from both subfamilies, and include seven sequence variants with different N- and C-terminal domains (Table S3). Precision measurements were conducted on 8 days over 4 weeks by three analysts. On each experimental day, each analyst independently grew (from separate frozen stocks), stained, acquired, and analyzed duplicates of the seven isolates, yielding a total of 48 determinations for each isolate. Each assay plate was read twice, once each using two Accuri C6 flow cytometers to assess variability in the reading process. The total percent relative standard deviation (RSD) for each of the seven isolates ranged from 13.2 to 29.1% (data not shown). Table 3 summarizes the results of variance component analysis for all of the isolates, which takes all factors, including day, analyst, cytometer, and replicates, into account. The percent total RSD for the panel of isolates was 21.2%, less than the 30% upper limit set as a prospective acceptance criterion in the validation.

10.1128/mBio.00036-18.9 TABLE S3  Properties of isolates used for MEASURE assay validation. Download TABLE S3, DOCX file, 0.02 MB. Copyright © 2018 McNeil et al. 2018 McNeil et al. This content is distributed under the terms of the Creative Commons Attribution 4.0 International license.

**TABLE 3:** Precision and repeatability of the MEASURE assay

Factor	Variance	SD	% RSD
Day	0.0007	0.0264	6.1
Analyst	0.0011	0.0334	7.7
Cytometers	0.0004	0.0196	4.5
Replicates	0	0	0.0
Residuals	0.0061	0.0782	18.2
Totals	0.0083	0.0912	21.2

### fHBP expression in NmB invasive-isolate set.

**FIG 4:**
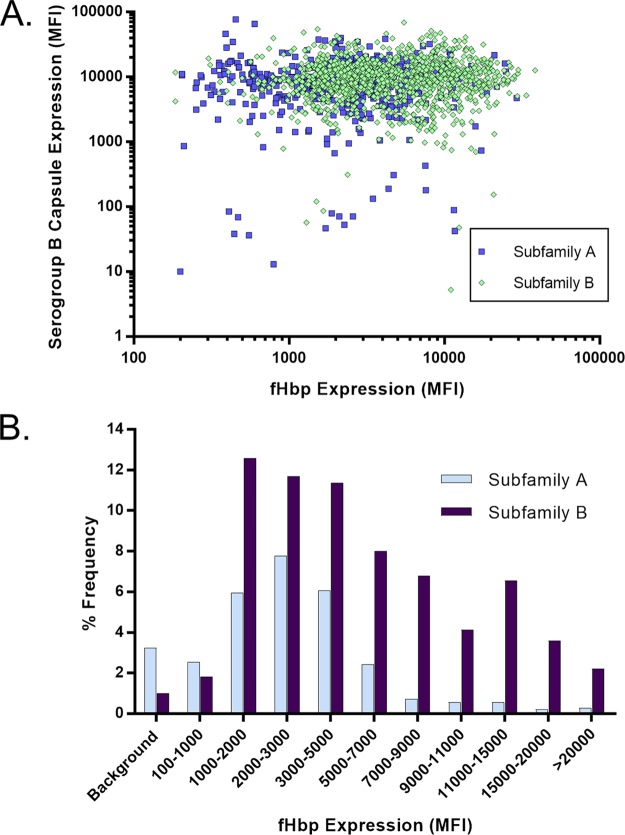
fHBP expression is detected on >95% of the isolates in the NmB invasive-isolate set (*n* = 1,814). fHBP surface expression levels of subfamily A (*n* = 550) and B (*n* = 1,264) isolates in the NmB invasive-isolate set (*n* = 1,814) are shown. (A) Correlation of serogroup B capsular polysaccharide MFIs (*y* axis) with the fHBP expression level MFI (*x* axis) as measured by the broadly cross-reactive fHBP MAb MN86-994-11-1. Subfamily A isolates are represented by blue squares, and subfamily B isolates are shown as green diamonds. (B) Isolates were binned on the basis of the level of fHBP expression (MFI) in the MEASURE assay. The *y* axis represents the frequency of isolates in each binned group. The background category includes strains with an MN86-994-11-1 MFI of <3 times the mouse IgG (negative control) MFI or an MFI of <100.

**FIG 5:**
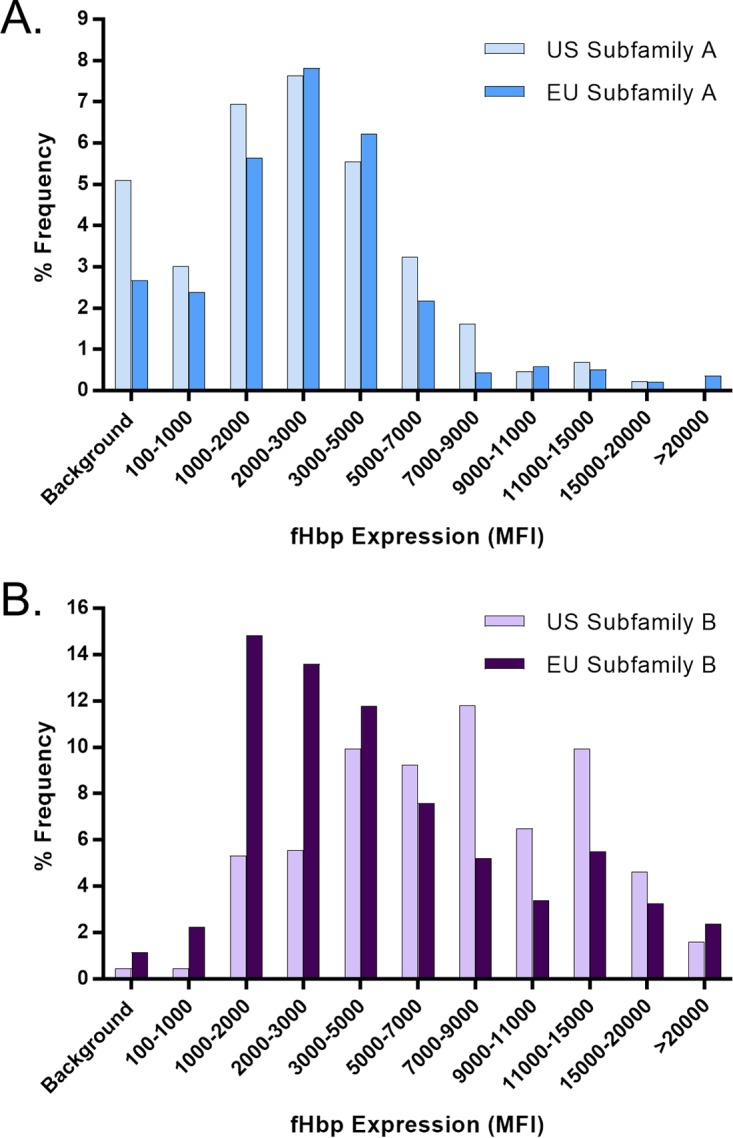
fHBP expression levels are distributed similarly in European Union (EU) and United States isolates. fHBP surface expression levels in the United States (*n* = 432) and European Union (*n* = 1,382) subsets of the prevalence-based collection of invasive NmB isolates (*n* = 1,814) are shown. (A) Subfamily A isolates from the United States (*n* = 149) and the European Union (*n* = 401). (B) Subfamily B isolates from the United States (*n* = 283) and the European Union (*n* = 981). Isolates were binned on the basis of their level of fHBP expression (MFI) in the MEASURE assay. The *y* axis represents the frequency of isolates in each binned group. Background denotes an MFI of <3 times the mouse IgG (negative control) MFI and/or an MFI of <100.

The MEASURE assay was used to assess the level of fHBP expression of 1,814 isolates in the NmB invasive-disease-causing isolate set that had been collected by a prospective systematic approach from the United States, the United Kingdom, France, Norway, Spain, Germany, and the Czech Republic (13, 26) (Table S1). The gene encoding fHBP was present in all 1,814 isolates. The fHBP expression profile of all 1,814 isolates was plotted according to serogroup B capsule expression or subfamily type with results displayed in aggregate and by geographic region or individual country (Fig. 4 to 5; Fig. S5 and S6). There was a wide range of fHBP expression levels among the isolates, with >50% of them expressing levels of fHBP corresponding to MFIs between 2,000 and 9,000. Approximately 95% of the isolates showed fHBP surface expression levels above the limit of detection (LOD) of the MEASURE assay, a level defined as at least an MFI of 100 and 3-fold the MFI observed with the mouse IgG experimental control antibody. Expression of serogroup B capsule had no impact on the ability to detect fHBP, as the antigen was as readily detected in isolates expressing the highest levels of capsular polysaccharide as those expressing lower levels with no discernible trend (Fig. 4A; Fig. S5). Median surface expression values were determined for the most prevalent fHBP subfamily A and B sequence variants in the NmB invasive-isolate set (A05, A07, A06, A12, A19, A22, B03, B09, B16, B24, B44), which account for >76% of the isolates in this collection (Table S4). Figure 5 shows that while the fHBP expression level distributions in subfamily A were very similar between isolates collected in the United States and Europe, the subfamily B expression level distribution tended to be higher in the United States, driven mostly by the preponderance of B24-expressing isolates (42% of the total) (27, 28), which generally have high fHBP surface expression levels (Table S4). A substantial proportion of these B24 United States isolates are from Oregon (104/184; 56.5%) where hyperendemic strains are circulating (29). Of the 77 NmB isolates below the assay LOD, 24 were United States isolates (24/432; 5.56%) and 53 were European Union isolates (53/1382; 3.84%). In both geographic regions, these isolates represented diverse sequence type (ST) clonal complexes and showed a similar age distribution (data not shown).

10.1128/mBio.00036-18.5 FIG S5  fHBP surface expression levels of NmB isolates in the invasive-isolate set (*n* = 1,814) by individual country as shown. Panels: A, United States isolates (*n* = 432); B, United Kingdom isolates (*n* = 536); C, French isolates (*n* = 244); D, Spanish isolates (*n* = 346); E, German isolates (*n* = 205). The correlation of capsule serogroup B MFIs (*y* axis) to fHBP expression level (MFI; *x* axis), as measured by broadly cross-reactive fHBP MAb MN86-994-11-1, is shown. Subfamily A isolates are represented by blue squares, and subfamily B isolates are represented by green diamonds. Download FIG S5, DOCX file, 0.1 MB. Copyright © 2018 McNeil et al. 2018 McNeil et al. This content is distributed under the terms of the Creative Commons Attribution 4.0 International license.

10.1128/mBio.00036-18.6 FIG S6  fHBP surface expression levels of NmB isolates in the invasive-isolate set (*n* = 1,814) by individual country as shown. Panels: A, United States isolates (*n* = 432); B, United Kingdom isolates (*n* = 536); C, French isolates (*n* = 244); D, Spanish isolates (*n* = 346); E, German isolates (*n* = 205). Isolates were binned on the basis of their fHBP expression (MFI) in the MEASURE assay. The *y* axis represents the frequency of isolates in each binned group. Background denotes an MFI of <3 times the mouse IgG (negative control) MFI and/or an MFI of <100. Download FIG S6, DOCX file, 0.5 MB. Copyright © 2018 McNeil et al. 2018 McNeil et al. This content is distributed under the terms of the Creative Commons Attribution 4.0 International license.

10.1128/mBio.00036-18.10 TABLE S4  fHBP expression medians in prevalent variants in the NmB invasive-isolate set. Variants with equal prevalences are indicated by asterisks. Download TABLE S4, DOCX file, 0.02 MB. Copyright © 2018 McNeil et al. 2018 McNeil et al. This content is distributed under the terms of the Creative Commons Attribution 4.0 International license.

### Predicting the probability of NmB isolate susceptibility in the hSBA.

While an hSBA is the only accepted surrogate of meningococcal vaccine efficacy, it was previously reported that surface expression of fHBP can predict susceptibility to killing of NmB isolates with fHBP immune serum samples, and a tentative threshold surface expression level associated with killing was proposed (15). In this more comprehensive investigation, we evaluated 109 NmB isolates by both MEASURE and an hSBA to confirm the relationship between the fHBP surface expression level and susceptibility to hSBA killing and to define a predictive MEASURE assay threshold based on statistical analysis. Sixty-four of these isolates were selected randomly and represent the genetic diversity of circulating invasive isolates identified in the prevalence-based strain collection (*n* = 1,814). In addition, some isolates selected from historical collections (*n* = 45) were also included. Collectively, these 109 isolates represent 12 diverse ST clonal complexes and expressed 38 different fHBP variants that account for 82.7% of the variants in the prevalence-based collection. The 22 subfamily A variants in this strain collection have ≥85.1% amino acid sequence identity with the variant A05 vaccine antigen, while the 16 subfamily B variants have ≥86.2% sequence identity with the variant B01 vaccine antigen. The levels of fHBP expression produced MFIs of 157 to 64,511, with a median MFI of 2,961.

**FIG 6:**
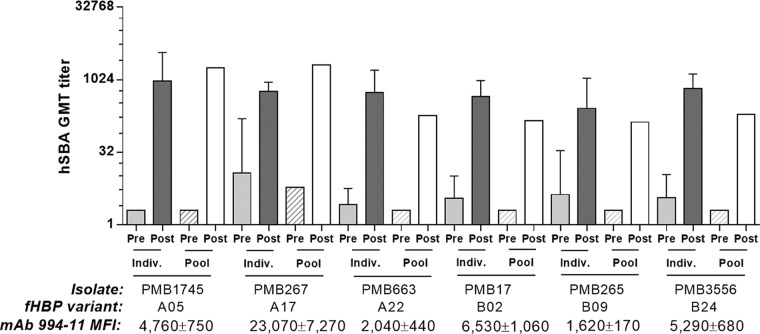
GMTs of individual serum samples from a phase 1 study are similar to those of serum pools in hSBAs with NmB isolates that express fHBP subfamily A or B variants. The susceptibility of subfamily A and B isolates to vaccine immune serum samples was evaluated, and the mean fHBP surface MFIs were determined by MEASURE assay with MAb MN86-994-11 (mean value of three experiments ± the standard deviation). Preimmune and immune serum samples from five individual subjects were pooled, and the hSBA activities of the pools were compared with those of individual serum samples (GMTs with 95% confidence intervals).

The activities of pre- and postvaccination serum samples from individual subjects were compared to a pool of the respective serum samples in hSBAs with 3 fHBP subfamily A and 3 subfamily B isolates selected from the 109 NmB strains. Results shown in Fig. 6 demonstrate that similar hSBA titers were obtained with postvaccination pooled serum samples and individual immune serum samples. This provided confidence that pooled serum samples could be used instead of individual serum samples for the testing of larger numbers of strains, thereby avoiding restriction of the scope of hSBA testing because of limited serum availability. Isolates with higher fHBP expression levels did not show significantly higher hSBA titers than those with lower expression levels, suggesting that beyond a certain threshold, isolates may be similarly susceptible to vaccine immune sera. The baseline hSBA titers of the prevaccination pooled serum samples were equal to or lower than the prevaccination geometric mean titers (GMTs) of the individual-subject serum samples comprising the pool. However, this had little impact on the fold increase over baseline responses (that define responder rates), as the postvaccination individual-subject serum GMTs were mostly (i.e., in four of six cases) slightly higher than the titers obtained with postvaccination pools.

**FIG 7:**
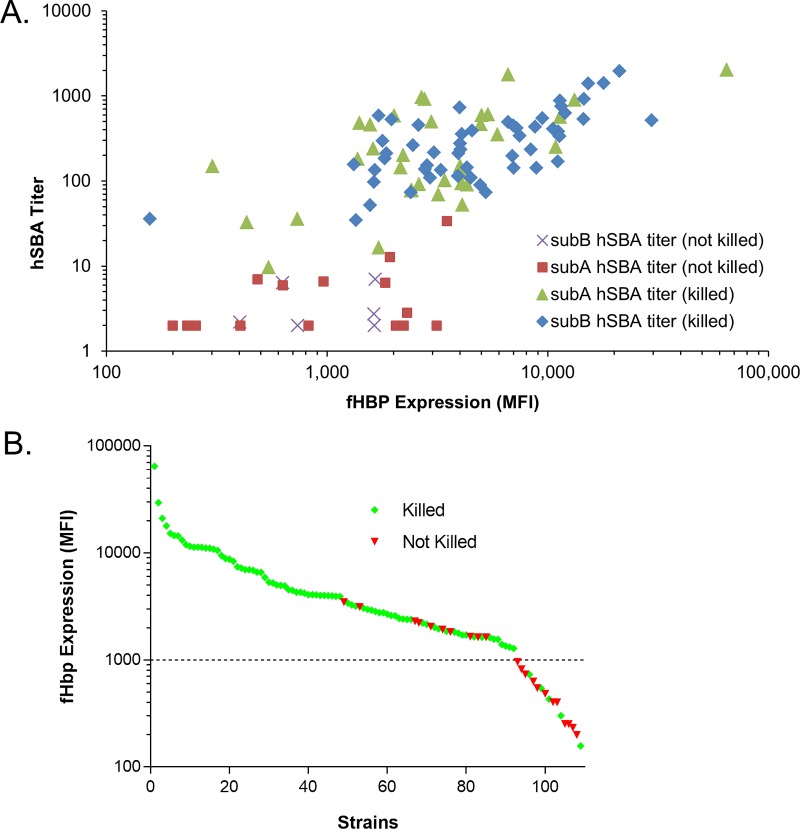
NmB fHBP surface expression level (MFI) and susceptibility to killing in an hSBA by bivalent fHBP immune serum. fHBP surface expression is determined by the binding of broadly cross-reactive MAb MN86-994-11-1 to NmB isolates in the MEASURE assay. The 109 NmB isolates were also tested in the hSBA with pooled human pre- and postvaccination bivalent rLP2086 immune serum samples. (A) For each of the 109 isolates, the postvaccination hSBA titer is plotted versus the level of fHBP surface expression. In cases where more than one assay was conducted with the same strain, the GMT was used. The symbol associated with each isolate conveys both the fHBP subfamily expressed and whether or not the isolate is killed (>4-fold increase in titer comparing pre- and postvaccination serum pools) with immune serum samples in an hSBA (B) Isolates were ordered from high to low fHBP surface expression and separated into killed and not killed groups on the basis of the ≥4-fold rise in hSBA titers between pre- and postvaccination serum pools. Green diamonds represent killed isolates, and red triangles represent isolates that were not killed. The dotted line marks the MFI threshold of 1,000.

Next, the pre- and postvaccination pooled serum samples were used to determine the susceptibility to killing in hSBAs by using the remainder of the isolates in the 109-strain set. Collectively, 87 (approximately 80%) of the 109 isolates were killed by bivalent rLP2086 vaccine-elicited antibodies in postvaccination serum (Fig. 7). A strain was considered susceptible in an hSBA only if a 4-fold rise in hSBA titer was observed between the pre- and postimmune human serum samples for >50% of the assays that met system suitability criteria with different complement lots (see Materials and Methods). For example, three isolates were killed in two out of five experiments (with different complement lots) but were not counted as susceptible. The median hSBA titer obtained for the group of 109 isolates with the pooled prevaccination serum was 2. Four strains yielded prevaccination hSBA titers of >8, and for these strains, 8- to 61-fold titer increases were observed in postvaccination serum samples. The median titer obtained by using the pooled postvaccination serum samples was 153. In hSBAs using the pooled postimmune serum samples, a titer of at least 8 was obtained for all of the isolates that were declared susceptible. There was a poor correlation (*R*^2^, <0.5) between the hSBA titer obtained and the level of fHBP detected in the MEASURE assay for strains expressing either subfamily A or subfamily B fHBPs (Fig. 7A; linear regression analysis of log-transformed data not shown). In contrast, visual inspection of the rank-ordered MFI data (Fig. 7B) for the 109 isolates evaluated in the hSBA suggested an MFI breakpoint of approximately 1,000, above which isolates were predominantly susceptible in an hSBA and below which a larger proportion of strains were not killed by bivalent rLP2086 postvaccination immune serum in the hSBA. Exceptions included 10/92 (11%) isolates above this cutoff that were refractory to bactericidal activity in an hSBA (7 subfamily A and 3 subfamily B isolates) and 5/17 (29%) isolates with fHBP surface expression levels below this cutoff that were susceptible (4 subfamily A isolates and 1 subfamily B isolate).

The probability that an NmB isolate randomly chosen from among those above a defined MFI fHBP expression cutoff will be susceptible in an hSBA was quantitatively determined by using a weighted analysis that adjusted the proportions of killed isolates for each variant by the prevalence of that variant in the NmB prevalence-based collection of 1,814 invasive isolates. The probabilities of such an isolate with an MFI above a threshold surface expression level being susceptible in an hSBA are shown in Table 4, along with the percentage of isolates within the set that were above the defined cutoff. According to this analysis, susceptibility in an *in vitro* hSBA can be predicted with high reliability for strains expressing fHBP at MFIs of >1,000.

**TABLE 4:** Probabilities of strain susceptibility in the prevalence-based collection of invasive NmB isolates

MFI cutoff	Probability of isolate with MFI above cutoff being killed (%)	% of isolates above cutoff		
		Total	Subfamily A	Subfamily B
500	87.4	96.9	92	99.1
1,000	91.2	91.4	80.9	96.0
1,100	91.2	90.4	79.5	95.2
1,500	90.3	84.6	73.6	89.4
2,000	95.8	72.8	61.1	77.9

## DISCUSSION

fHBP is an important virulence factor for *N. meningitidis* that protects the bacteria from the innate immune response by downregulating the alternative complement pathway (10, 30, 31). While fHBP expression cannot be directly measured *in vivo*, patients recovering from NmB infections develop anti-fHBP antibodies during convalescence (32). In addition, anti-fHBP antibodies are acquired with age, presumably through carriage acquisition, and are found at levels similar to those in patients recovering from invasive disease (32, 33). These data provide evidence that fHBP is expressed *in vivo* in humans during carriage and invasive disease. fHBP expression can be detected *in vitro* by the flow cytometric MEASURE assay. NmB isolates are grown in liquid culture suspension (rather than agar medium) to better mimic the conditions in the blood during infection. Furthermore, the bacteria are left intact (rather than lysed) to determine both fHBP surface expression and the accessibility of fHBP to antibodies in the presence of other surface antigens, including capsular polysaccharide, providing a context similar to that encountered by the immune system during infection. The MEASURE assay employs a broadly cross-reactive MAb, MN86-994-11-1, that recognizes isolates expressing any fHBP variant. Deuterium-hydrogen exchange mapping and targeted mutagenesis demonstrated that this MAb binds to a conformational epitope in the C-terminal β-barrel domain conserved in fHBP variants from both subfamilies. The binding of this MAb to homologous structural segments within both subfamilies of fHBP is essential for the ability of the MEASURE assay to accurately quantitate the level of fHBP expressed on the cell surface of all NmB isolates, regardless of the subfamily or variant. In summary, a combination of *in vitro* binding kinetics, flow cytometry, epitope mapping, and fHBP sequence data confirms that the MN86-994-11-1 binding site is conserved among fHBP antigen variants and that it is accessible on the bacterial surface to the host immune system.

The MEASURE assay was validated and shown to be a specific and reproducible flow cytometry-based method used to detect the level of fHBP expressed on the bacterial surface. In a large, prevalence-based collection of invasive NmB isolates (*n* = 1,814) from national reference laboratories in the United States and Europe, 95.8% of them demonstrated fHBP expression levels greater than the LOD of the assay (i.e., an MFI of at least 100 and greater than three times the MFI observed when using the negative-control IgG). Overall, fHBP expression levels range from the LOD to an MFI of >50,000. The majority of isolates have moderate expression of fHBP (MFIs of 2,000 to 9,000), with a median value of 3,300, which corresponds to approximately 120 pg of fHBP/µg of cell lysate. NmB isolates expressing fHBP levels that are below the LOD are not associated with any particular genotype (as assessed by ST); rather, multiple clonal complexes are represented. Although it was not investigated in this study, previous analysis indicates that the fHBP expression level may be influenced by the sequence of the fHBP promoter region (34). Another contributing factor may be the lower stability and greater flexibility of fHBP subfamily A variants, revealed by hydrogen-deuterium exchange mapping and isothermal scanning calorimetry experiments, which may lead to faster protein degradation and turnover.

Since antibody cross-linking is important for complement deposition and bacterial killing, sufficient fHBP expression levels are an important indicator of bacterial isolate susceptibility. To this end, the MEASURE assay was used to characterize the fHBP expression levels of all available isolates and further of each fHBP variant group. An isolate with very low fHBP expression may not be susceptible to antibody cross-linking and subsequent formation of the complement-mediated membrane attack complex required for killing of the isolate and thus would not be appropriate for use in an hSBA. In contrast, one could perceive that an isolate with extremely high fHBP expression may be more easily killed in an hSBA and not truly represent the preponderance of NmB invasive-disease-causing isolates within each fHBP variant group. In the present analysis, we demonstrate that isolates expressing fHBP at the low end of the spectrum (MFI of <1,000 or <30 pg of fHBP/μg of cell extract) can still be killed in an hSBA by bivalent rLP2086 immune serum samples. This is in contrast to the findings of Biagini et al. (34), who determined that serum samples elicited by the multicomponent 4CMenB vaccine, which includes a single nonlipidated fHBP antigen, could not kill strains that expressed <135 pg of fHBP/μg of cellular extract. This difference could be due to the absence of a lipidated fHBP in 4CMenB, as lipidated fHBPs present in bivalent rLP2086 vaccine induce a substantially more potent immune response in preclinical studies than that of their nonlipidated counterparts (22). It is important to note the two major differences between the 4CMenB assessment of Biagini et al. and that described here. First, the 4CMenB assessment was done with murine serum samples, not human serum samples, as the bivalent rLP2086 assessment was, so one cannot relate the former results to clinical outcomes. Second, the 4CMenB assessment was conducted in SBAs using rabbit complement (rSBA). The accepted SBA format for predicting vaccine efficacy against serogroup B meningococci uses human complement, as rabbit complement does not provide specificity, resulting in higher titers, which may lead to overestimation of the vaccine’s effect (35, 36); this is contrary to the finding of lower sensitivity with 4CMenB murine serum in an rSBA than in the studies reported here with human bivalent rLP2086 serum in an hSBA.

Despite determining that some strains with low levels of fHBP were susceptible to immune serum in an hSBA, we sought to identify a predictive threshold where most strains were susceptible. A probability approach was used to estimate whether strains with specified levels of fHBP would be susceptible in an hSBA to the bactericidal antibodies elicited by bivalent rLP2086. An MFI of 1,000 (approximately 30 pg/μg of NmB whole-cell extract) appeared to define a natural breakpoint where the majority of strains with fHBP MFIs of >1,000 were found to be susceptible in an hSBA using serum obtained from young adults immunized with bivalent rLP2086. Ten of 92 isolates that expressed fHBP at levels above the selected predictive threshold were defined as refractory to hSBA killing. These included strains that expressed fHBP variants A05, A12, A19, A22, A47, B01, and B16. Other isolates expressing most of these fHBP variants can be reproducibly killed in hSBAs (23), suggesting that the inability of these isolates to be killed in an hSBA may be due to cell surface factors other than fHBP. Other surface components implicated in resistance to serum-mediated bactericidal activity include sialylated lipopolysaccharides and TspB proteins (37–39). In practice, such interference may reflect a common challenge in hSBA development, which is the difficulty, for some isolates, in identifying conditions that can induce killing in the presence of a vaccine-elicited antibody. Although it is impractical, isolate susceptibility to a vaccine-elicited antibody in such cases can be assessed in bactericidal assays with homologous complement (non-heat-killed prevaccination serum samples from the same subject) or whole blood.

Our statistical analysis predicts that 91% of the isolates in the set of 1,814 invasive NmB isolates express fHBP at sufficient levels to be susceptible to bactericidal killing by rLP2086 vaccine-induced antibodies (i.e., MFIs of >1,000 or approximately 30 pg of fHBP/μg of NmB protein). Given that some isolates below the defined fHBP threshold can still be shown to be susceptible to human postvaccination polyclonal serum in an hSBA, the MEASURE assay may underestimate the potential NmB isolate coverage by the vaccine. Overall, this assessment illustrates the potential of this vaccine to provide broad coverage against diverse invasive-disease-causing NmB isolates.

Bivalent rLP2086 was licensed by demonstrating that it generated antibodies that could be shown in an hSBA to kill NmB isolates representative of isolates circulating in the United States and Europe. The MEASURE assay was used to ensure the selection of representative NmB isolates for use in hSBAs for the clinical studies. Four diverse epidemiologically relevant NmB isolates that expressed fHBP variants heterologous to the vaccine variants were selected in an unbiased fashion from the 1,814-invasive-strain set, adjusted for prevalence, and tested in the hSBA to assess the efficacy and breadth of isolate coverage. Clinical study endpoints were achieved with these four strains in hSBAs with serum samples from vaccinated subjects (reviewed in reference 24). Similar performance in hSBAs has been demonstrated with additional isolates selected to be representative of fHBP variant groups and for isolates that have recently caused meningococcal serogroup B outbreaks (23, 40).

The MEASURE assay was developed as a flow cytometric analytic assay to measure the native surface expression of fHBP variants on meningococci. We find that expression levels and the proportion of strains above the level associated with susceptibility in an hSBA are generally consistent across NmB isolates from the United States and Europe. The assay has been transferred to two national reference laboratories (Public Health England and the Centers for Disease Control and Prevention [CDC]), and interlaboratory evaluations are planned. Though the assay can be used to predict which NmB isolates are potentially susceptible in the hSBA to bactericidal activity elicited by bivalent rLP2086, knowledge of the fHBP expression level for a given strain cannot predict the percentage of human subjects who demonstrate bactericidal activity following vaccination and thus the efficacy of the vaccine in the general population. Until vaccination with NmB vaccines is implemented broadly, population level coverage conferred by them can only be inferred through the use of hSBAs and assessment of serum samples from individual vaccinees.

## MATERIALS AND METHODS

### NmB invasive-isolate set and other strains.

To form the NmB invasive-isolate set (*n* = 1,814), invasive NmB isolates were systematically obtained from public health laboratories from 2000 to 2006 in the United States, the United Kingdom, France, Norway, and the Czech Republic (*n* = 1,263) as described by Murphy et al., with the addition of isolates from Spain and Germany (*n* = 551) (Table S1) (11, 13). A 45-invasive-strain collection obtained mostly prior to the year 2000 from the United States and Europe was also used. Sixty-four invasive isolates randomly selected from a 1,263-isolate subset of the 1,814-invasive-isolate set plus the 45-strain collection composed the 109-strain set used in hSBAs. United States isolates were obtained from the CDC Active Bacterial Core Surveillance (ABCs) bacterial collection. Strains lacking the gene coding for fHBP, for use as flow cytometry negative controls, were constructed by insertional inactivation of the open reading frame as described previously (9).

### Growth of NmB isolates.

NmB isolates were grown as previously described (41). Briefly, bacteria were grown to an optical density at 600 nm between 0.50 and 0.55 and subsequently fixed in 1% (vol/vol) paraformaldehyde (PFA) in 1× phosphate-buffered saline (PBS) for 10 min to 3 h at 4°C. The fixed bacterial samples were kept on ice until staining.

### Generation of MAb MN86-994-11-1.

Hybridomas were generated as previously described (41). The MN86-994-11 antibody was identified from a hybridoma fusion derived from mice vaccinated with rLP2086-A05. Parental hybridoma cultures were initially screened for reactivity against homologous rLP2086 protein from strain PMB1745 (A05 variant), heterologous rLP2086 protein from strain PMB1135 (B01 variant), and whole cells of *N. meningitidis* strains expressing fHBP variant A05 or B01. Of the 165 reactive parent hybridomas identified from this fusion, culture supernatants from 33% (*n* = 54) showed cross-reactivity with the heterologous fHBP-B01 antigen or strain. Screening of candidate hybridomas from 10 other fusions with fHBP variants as immunogens yielded a total of 97 parents showing some level of cross-reactivity with antigens from both subfamilies A and B. The broadly cross-reactive hybridoma parent MN86-994 was cloned by single-cell limiting dilution, and the clone isotype was confirmed by Roche IsoStrips to be IgG2b κ.

### Recombinant antigens.

Nonlipidated recombinant fHBP (rP2086) variants were expressed and purified as previously described (25). Mutations in the MN86-994-11 binding epitope were introduced by site-directed mutagenesis. In this case, a His-tagged version of rP2086-B01 cloned into plasmid vector pET30a was used as the mutagenesis template to facilitate the purification of recombinant mutants. A mutagenesis kit was used in accordance with the manufacturer’s instructions (QuikChange; Agilent), mutagenic oligonucleotides used in the reaction were designed with the QuikChange Primer Design Program, and the presence of intended mutations and the absence of secondary mutations were confirmed by DNA sequencing. Mutant proteins expressed in *Escherichia coli* BL21(DE3) were purified by Ni Sepharose affinity chromatography and size exclusion chromatography (GE Healthcare). All CD and ITC experiments were done with 1× PBS, pH 7.4. Protein and antibody samples were thoroughly dialyzed against experimental buffer. Concentrations of rP2086-B01 and MN86-994-11 were determined spectrophotometrically by using extinction coefficients of 0.363 and 1.4 (mg/ml)^−1^ cm^−1^ at 280 nm, respectively. Light scattering was taken into account as previously described (42).

### Affinity measurements.

The affinities of multiple rP2086 (fHBP) variants for MN86-994-11-1 were tested with the Octet RED96 System (FortéBio). Nonlipidated recombinant fHBP variants A05, A62, A29, A22, B44, B24, B22, and B01 were individually biotinylated in a one-step reaction with an *N*-hydroxysuccinimide–PEG4–biotin kit. These variants were chosen to represent antigens with a high degree of sequence divergence from the A05 and B01 vaccine antigens. The biotinylated fHBP antigens were adsorbed to streptavidin biosensors, and MAb MN86-994-11-1 association and dissociation rates were determined. The protein concentration used was 10 µg/ml, and seven 2-fold serial dilutions of the MN86-994-11-1 MAb, starting at 25 nM, were used. The raw data were collected and analyzed with Octet Software.

### Western immunoblot assay.

Cell pellets of meningococcal bacteria were lysed, and the total cell protein concentration was determined by the Peterson-modified Lowry protein assay (43). Proteins were separated by SDS-PAGE and then blotted onto nitrocellulose membrane. A standard curve was also generated with purified recombinant fHBP of each variant loaded onto the gel in the range of 31.25 to 8,000 pg. The blots were blocked with 5% skim milk in 1× PBS and then incubated with rabbit anti-bivalent fHBP polyclonal antibodies. After being washed, the blots were incubated in secondary antibody, goat anti-rabbit IgG (H+L) Cy5. Imaging was performed with a 633-nm laser with a 670/BP30 band-pass filter. The images were then analyzed with ImageQuant v5.2 software.

### Hydrogen-deuterium exchange experiments.

The general operational procedures and HDX-MS apparatus used have been previously described in detail (44–48). Proteolytic digestion, high-performance liquid chromatographic separation, mass spectrometric analysis, and peptide identification were done exactly as previously described, with rP2086-A05 or rP2086-B01 protein and MAb Mn86-994-11-1 (49). The amount of deuterium accumulated was determined from changes in the molecular weights of the corresponding peptides at various time points as previously described (49).

### CD spectroscopy.

All CD experiments were done on a Jasco J-810 automated recording spectropolarimeter equipped with a Jasco PTC-424S six-position Peltier-type cell holder. Near-UV CD spectra were recorded at 20°C in 1-cm rectangular quartz cells from 250 to 320 nm at 100 nm/min, a data pitch of 0.1 nm, and a bandwidth of 3 nm. The protein concentration was 1.0 mg/ml. Five accumulations were collected and averaged per spectrum. Data were corrected for baseline contributions and smoothed with the SpectraManager software provided by Jasco. Near-UV CD data are reported as molar ellipticity, which was calculated with the equation θ_molar_ = θ/(10 ⋅ *l* ⋅ *C*), where θ_molar_ is the molar ellipticity (millidegrees × square centimeters/decimole), θ is the experimentally measured ellipticity in millidegrees, *l* is the cell path length in centimeters, and *C* is the protein molar concentration.

Far-UV CD spectra were recorded from 200 to 260 nm in 1-mm rectangular quartz cells, with other parameters being identical to the near-UV CD experiments. The protein concentration was 0.33 mg/ml. Far-UV CD data are reported as mean residue ellipticity, which was calculated with the equation θ_MRE_ = (θ × 106.9)/(10 ⋅ *l* ⋅ *C*), where θ_MRE_ is the mean residue ellipticity (millidegrees × square centimeters/decimole), θ is the experimentally measured ellipticity in millidegrees, *l* is the cell path length in centimeters, and *C* is the protein concentration in milligrams per milliliter.

### ITC.

ITC experiments were done with a VP-ITC microcalorimeter (Microcal, Northampton, MA) at 25°C. All samples were centrifuged at 14,000 rpm for at least 10 min and thoroughly degassed. MN86-994-11 antibody solutions (4.1 μM) were titrated with 123 to 154 µM solutions of individual MnB rP2086-B01 variants. An initial 2-µl injection was followed by 7-µl injections at 180-s intervals until no heat exchanges were observed. MN86-994-11 titrations were corrected for the heat of protein dilution by performing buffer titrations with the same protein solutions under identical conditions and subtracting the results from the antibody titrations. Data were fitted to the “single class of binding sites” model with VP-ITC data analysis software provided by Microcal to determine the binding stoichiometry (*N*), association constant (*K*_*a*_), enthalpy change (Δ*H*), and entropy change (Δ*S*) upon binding. The dissociation constant (*K*_*d*_) was calculated as 1/*K*_*a*_.

### MEASURE assay.

A volume of 50 μl of bacteria fixed in 1% PFA–PBS was plated per well of 96-well U-bottom polystyrene plates, centrifuged, and washed once in 1% (wt/vol) bovine serum albumin in 1× PBS. MAb MN86-994-11-1 or mouse IgG (negative control) was added to the bacterial pellets, which were resuspended and incubated on ice for 30 min. After two washes, biotinylated goat anti-mouse IgG (subclasses 1, 2a, 2b, and 3; Jackson ImmunoResearch, Inc.) was added to the cell pellets, which were resuspended and incubated on ice for 30 min. The cells were washed twice and resuspended in streptavidin-phycoerythrin (PE; BD Biosciences) and incubated on ice for 30 min. After an additional two washes, the cell pellets were resuspended in 1% PFA. Twenty thousand events per well were acquired on an Accuri C6 flow cytometer and analyzed with Accuri CFlow software (see Fig. S1 for the gating strategy used). The MFI of the PE channel was determined for each sample after gating on bacterial cells in the logarithmic forward scatter versus side scatter dot plot. For fHBP expression to be considered above the LOD of the MEASURE assay, MFIs had to be above an arbitrary threshold of at least 100 and three times that of the control mouse IgG MFI in that assay. Serogroup B capsular expression was determined by the same staining procedure as previously described, with the exception of the use of an anti-serogroup B MAb (NIBSC), followed by incubation with biotinylated goat anti-mouse IgM (Southern Biotech).

### hSBA.

hSBAs with human serum samples from young adults were performed as previously described (19, 50, 51). Human serum with no intrinsic detectable bactericidal activity in screening assays was used as the exogenous complement source. Subject-matched pooled preimmune serum samples were used to demonstrate that the hSBA titers observed in the pooled postimmune serum samples were the result of vaccine-induced antibodies. Moreover, depletion experiments were performed to demonstrate the specificity of the antibodies for fHBP. Briefly, fHBP from the same subfamily competed with the binding of serum antibodies with antigen expressed on the surface of the bacteria and significantly reduced the hSBA titers, whereas irrelevant proteins and polysaccharides used as competitors did not (data not shown). In the study reported here, 45 of the 109 NmB strains were tested with pre- and postimmune serum samples from five subjects enrolled in young adult clinical study 6108A1-500 (18 to 25 years old) and 64 NmB strains were tested with pre- and postimmune serum samples from four of the same five subjects because insufficient serum was available from the fifth subject (20). Strains were tested in hSBAs with the pooled human serum samples and up to five human serum complement lots. A strain tested in the hSBA was designated killed if a 4-fold rise in the hSBA titer was observed between the pre- and postimmune human serum samples in >50% of the assays that met system suitability criteria. This stringent approach was taken so that strains could be identified that could be used for clinical testing. In some instances, strains that could be killed by bivalent rLP2086 serum were scored negative as they could only be killed with specific complement sources. Appropriate system suitability was achieved if the ratio of the number of surviving bacteria after the bactericidal incubation in the absence of serum samples (*T*_30_) to the number of input bacteria (*T*0) was ≥50%. A strain was considered not susceptible (not killed) in the hSBA if a 4-fold rise in the hSBA titer was not observed in >50% of the assays. As an example, if the hSBA titer of the preimmune serum pool was <1:4 (or a titer of 2) for a given NmB strain, then an hSBA titer of ≥1:8 with the postimmune serum pool would be required to achieve a 4-fold rise; if such a 4-fold rise was observed for >50% of the assays (e.g., two or three of three assays meeting system suitability criteria, three or four of four assays meeting system suitability criteria, three to five of five assays meeting system suitability criteria), a given NmB strain would be considered susceptible (killed) in the hSBA.

### Statistical analysis.

For the hSBA strain pool (*n* = 109), the numbers and proportions of strains below and above possible cutoffs of strains killed in the hSBA were determined. However, because the strains tested in the hSBA strain pool were not chosen to mimic the proportions of fHBP variants in the larger, more representative, extended NmB strain pool (*n* = 1,814), a weighted analysis was performed by adjusting the proportions of killed strains for each variant by the prevalence of that variant in the extended NmB strain pool. For strains above the cutoff, *P*(strain killed by hSBA) = ∑*_i_P*(strain killed by hSBA given that strain is variant type *i*) × *P*(strain is variant type *i*). The first factor in each term of this sum was estimated from the human SBA strain pool (*n* = 109) data. The second factor, the weight, was estimated from the extended NmB strain pool (*n* = 1,814).
