# Supplementary material for: Predicting the Susceptibility of Meningococcal Serogroup B Isolates to Bactericidal Antibodies Elicited by Bivalent rLP2086, a Novel Prophylactic Vaccine
Source: mBio. 2018 Mar 13;9(2):e00036-18. doi: 10.1128/mBio.00036-18 (PMC5850321; doi:10.1128/mBio.00036-18)
Supplement: FIG S1 [file mbo001183767sf1.docx]

**Supplemental Figure S1.** Gating Strategy for MEASURE assay. Representative gating strategy of a *N. meningitidis sample* stained with either MN86-994-11-1 or mouse IgG (negative control). A) FSC versus SSC dot plot on the log scale with a liberal gate set around the bacterial population. B) Histogram overlay of MN86-994-11 (aqua) and mIgG (red).
