# Supplementary material for: Predicting the Susceptibility of Meningococcal Serogroup B Isolates to Bactericidal Antibodies Elicited by Bivalent rLP2086, a Novel Prophylactic Vaccine
Source: mBio. 2018 Mar 13;9(2):e00036-18. doi: 10.1128/mBio.00036-18 (PMC5850321; doi:10.1128/mBio.00036-18)
Supplement: FIG S2 [file mbo001183767sf2.docx]

**Supplemental Figure S2.** Temperature-induced unfolding of fHBP monitored by DSC reveals that the N-terminal domain of fHBP-A05 denatures at a substantially lower temperature than fHBP-B01. All experiments were done on a VP-DSC differential scanning microcalorimeter in 1x PBS, pH 7.4 at 1.3-1.4 mg/ml protein concentration. Data were corrected for the instrumental baseline and normalized by protein concentration using Origin 6.0 software provided by the DSC manufacturer. Melting temperatures (T_m_) were defined as maxima of the individual unfolding transitions.
