# Supplementary material for: Predicting the Susceptibility of Meningococcal Serogroup B Isolates to Bactericidal Antibodies Elicited by Bivalent rLP2086, a Novel Prophylactic Vaccine
Source: mBio. 2018 Mar 13;9(2):e00036-18. doi: 10.1128/mBio.00036-18 (PMC5850321; doi:10.1128/mBio.00036-18)
Supplement: FIG S3 [file mbo001183767sf3.docx]

**Supplemental Figure S3.** An N-terminal domain peptide of fHBP-B01 is less susceptible to deuterium exchange due to higher intrinsic stability than the homologous peptide from fHBP-A05. It is impossible to say, therefore, whether or not this structural segment is involved in mAb-994-11 binding to fHBP-B01. Representative deuterium uptake plots for the proteolytic peptides derived from the N-terminal domains of fHBP proteins. Y-axes – number of protons exchanged for deuterium at each time point, x-axes -- incubation time. Blue symbols and lines – deuterium uptake by the isolated proteins, red lines – deuterium uptake by the proteins in the presence of mAB-994-11. Peptides are identified by the position of the first and last residue within the amino acid sequence of the protein (i.e., peptide 98-112 covers residues 98-112).


**Supplemental Figure S4.** Circular dichroism spectra of rP2086-B01 variants. ***A*** – far-UV CD spectra (secondary structural information), ***B*** – near-UV CD spectra (tertiary structural information). Spectra of individual variants are shown in different colors, as indicated.

**A**

**B**
