# Supplementary material for: Predicting the Susceptibility of Meningococcal Serogroup B Isolates to Bactericidal Antibodies Elicited by Bivalent rLP2086, a Novel Prophylactic Vaccine
Source: mBio. 2018 Mar 13;9(2):e00036-18. doi: 10.1128/mBio.00036-18 (PMC5850321; doi:10.1128/mBio.00036-18)
Supplement: FIG S4 [file mbo001183767sf4.docx]

**Supplemental Figure S4.** Circular dichroism spectra of rP2086-B01 variants. A) far-UV CD spectra (secondary structural information), B) near-UV CD spectra (tertiary structural information). Spectra of individual variants are shown in different colors, as indicated.

**A**

**B**
