# Supplementary material for: Predicting the Susceptibility of Meningococcal Serogroup B Isolates to Bactericidal Antibodies Elicited by Bivalent rLP2086, a Novel Prophylactic Vaccine
Source: mBio. 2018 Mar 13;9(2):e00036-18. doi: 10.1128/mBio.00036-18 (PMC5850321; doi:10.1128/mBio.00036-18)
Supplement: TABLE S1 [file mbo001183767st1.docx]

**Supplemental Table S1. NmB invasive Isolate set**

| Region | Number of Isolates | Approx. % Disease  Coverage In the  Starting  Regional Pool* | fHBP Subfamily (%) | |
| --- | --- | --- | --- | --- |
|  |  |  | Subfamily A | Subfamily B |
| US (ABCs sites) | 432 | 13 | 35 | 65 |
| UK countries† | 536 | 90 | 23 | 77 |
| France | 244 | 80-85 | 32 | 68 |
| Norway | 23 | 85-90 | 35 | 65 |
| Czech Republic | 28 | 50-70 | 25 | 75 |
| Spain | 346 | 70-85 | 40 | 60 |
| Germany | 205 | 70 | 21 | 79 |
| Total | 1814 |  | 30 | 70 |

* The regional ABCs sites cover ~13% of US population, and all isolates are included in the pool. European collections survey the entire country, and every 8^th^ isolate (12.5%) from the available collection of each country is included in the pool. † England, Wales, Northern Ireland.
