# Supplementary material for: Predicting the Susceptibility of Meningococcal Serogroup B Isolates to Bactericidal Antibodies Elicited by Bivalent rLP2086, a Novel Prophylactic Vaccine
Source: mBio. 2018 Mar 13;9(2):e00036-18. doi: 10.1128/mBio.00036-18 (PMC5850321; doi:10.1128/mBio.00036-18)
Supplement: TABLE S2 [file mbo001183767st2.docx]

**Supplemental Table S2. Titration of mAb MN86-994-11-1 against Subfamily A and B variants**

|  | **fHBP Variant** | | | | | | |  |
| --- | --- | --- | --- | --- | --- | --- | --- | --- |
|  | **A04** | **A05** | **B03** | **B09** | **B09** | **B09** | **B16** |  |
| **Conc. (μg/ mL)** | **Mean Fluorescence Intensity (MFI)** | | | | | | |  |
| 40 | 7136 | 6320 | 6844 | 4339 | 4563 | 7019 | 5272 |  |
| 20 | 7208 | 7541 | 6758 | 4900 | 4732 | 6300 | 5233 |  |
| 13.3 | 7035 | 6540 | 6726 | 4494 | 4656 | 5889 | 5064 |  |
| 10 | 6708 | 6208 | 6597 | 4828 | 4607 | 5457 | 5049 |  |
| 8 | 6448 | 5956 | 6384 | 4801 | 4393 | 5411 | 4936 |  |
| 6.7* | 6165 | 5803 | 6370 | 4751 | 4287 | 5120 | 4866 |  |
| 5.7 | 5694 | 6067 | 6223 | 4092 | 4137 | 4860 | 4694 |  |
| 5 | 5479 | 5696 | 5970 | 4476 | 3944 | 4858 | 4488 |  |
| 2 | 3776 | 4920 | 5130 | 3601 | 3112 | 3796 | 3730 |  |

* Shaded row represents the fHBP MFI determined at the concentration of MN86-994-11-1 used in the MEASURE assay
