# Supplementary material for: Predicting the Susceptibility of Meningococcal Serogroup B Isolates to Bactericidal Antibodies Elicited by Bivalent rLP2086, a Novel Prophylactic Vaccine
Source: mBio. 2018 Mar 13;9(2):e00036-18. doi: 10.1128/mBio.00036-18 (PMC5850321; doi:10.1128/mBio.00036-18)
Supplement: TABLE S3 [file mbo001183767st3.docx]

**Supplemental Table S3.** Properties of isolates used for MEASURE assay validation

| Strain | Yr of Isolation | Patient Age (yr) | fHBP variant | fHBP subgroup | % AA Identity (A05/B01) | PorA subtype | ST | CC | Measure GeoMean  994_11^†^ | Measure GeoMean  mIgG^†^ |
| --- | --- | --- | --- | --- | --- | --- | --- | --- | --- | --- |
| PMB2802* | 2005 | 11-25 | A22 | N2C2 | 88.9 | P1.19,15-1 | 43 | ST-41/44 complex/Lineage 3 | 11688 | 203 |
| PMB1745* | 1998 | NA | A05 | N1C2 | 100 | P1.22,14 | 2100 | ST-213 complex | 6601 | 226 |
| PMB3536* | 2001 | 7 | B03 | N6 | 90.8 | P1.4,15 | 41 | ST-41/44 complex/Lineage 3 | 3274 | 171 |
| PMB3242 | 2002 | 1-10 | B16 | N6 | 86.2 | P1.7-2,4 | 4489 | ST-41/44 complex/Lineage 3 | 1950 | 188 |
| PMB1135 | <2000 | NA | B01 | N5 | 100 | P1.7-1,1 | 44 | ST-41/44 complex/Lineage 3 | 1631 | 189 |
| PMB2058* | 2003 | >25 | A12 | N2C1 | 85.4 | P1.7-2,13-25 | 2976 | ST-269 complex | 2293 | 187 |
| PMB3453 | 2002 | <1 | A19 | N2C2 | 88.1 | P1.22-1,14 | 35 | ST-35 complex | 960 | 177 |

* isolates tested by quantitative Western blot

^†^Values are from pre-validation assay

NA – information not available
