# Supplementary material for: Predicting the Susceptibility of Meningococcal Serogroup B Isolates to Bactericidal Antibodies Elicited by Bivalent rLP2086, a Novel Prophylactic Vaccine
Source: mBio. 2018 Mar 13;9(2):e00036-18. doi: 10.1128/mBio.00036-18 (PMC5850321; doi:10.1128/mBio.00036-18)

**Supplemental Figure S5.** fHBP surface expression levels for NmB isolates in the invasive isolate set (n=1814) by individual country as shown. A) US isolates (n=432). B) UK isolates (n=536). C) French isolates (n=244). D) Spanish isolates (n=346). E) German isolates (n=205). Correlation of capsule serogroup B mean fluorescence intensity (MFI) values (y-axis) to fHBP expression level MFI (x-axis) as measured by broadly cross-reactive fHBP mAb MN86-994-11-1, Subfamily A isolates are blue squares and Subfamily B isolates are shown as green diamonds.


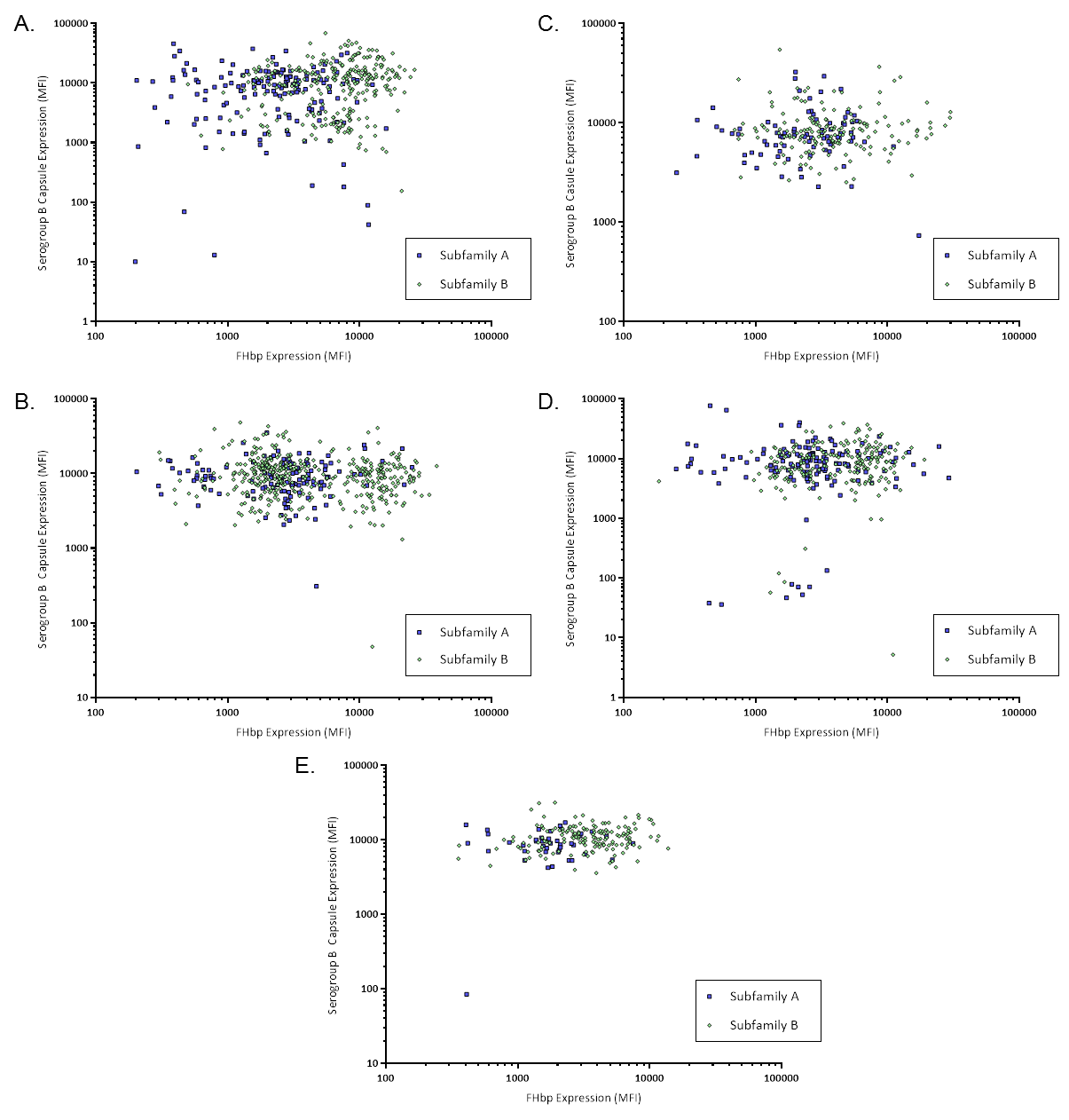

Supplement: FIG S5 [file mbo001183767sf5.docx]
