# Supplementary material for: Predicting the Susceptibility of Meningococcal Serogroup B Isolates to Bactericidal Antibodies Elicited by Bivalent rLP2086, a Novel Prophylactic Vaccine
Source: mBio. 2018 Mar 13;9(2):e00036-18. doi: 10.1128/mBio.00036-18 (PMC5850321; doi:10.1128/mBio.00036-18)

**Supplemental Figure S6.** fHBP surface expression levels for NmB isolates in the invasive isolate set (n=1814) by individual country as shown. A) US isolates (n=432). B) UK isolates (n=536). C) French isolates (n=244). D) Spanish isolates (n=346). E) German isolates (n=205). Isolates were binned based on their fHBP expression (MFI) in the MEASURE assay. The Y-axis represents the frequency of isolates in each binned group. Background denotes an MFI of less than 3 times the mouse IgG (negative control) MFI and/or an MFI of less than 100.

A.

C.

E.

D.

B.


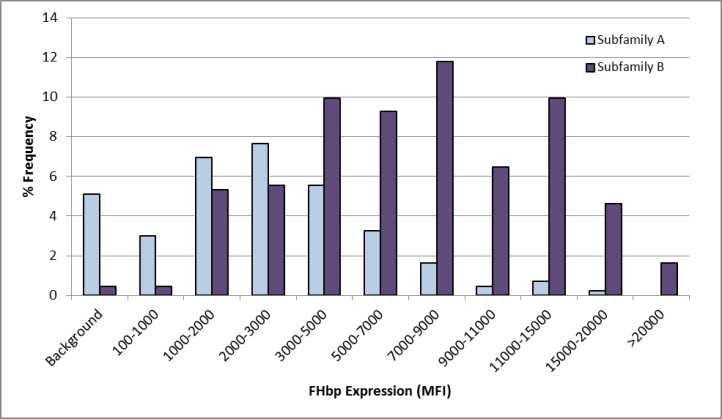

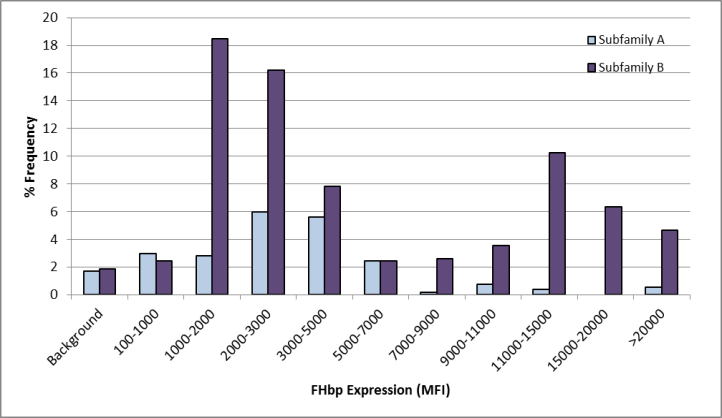

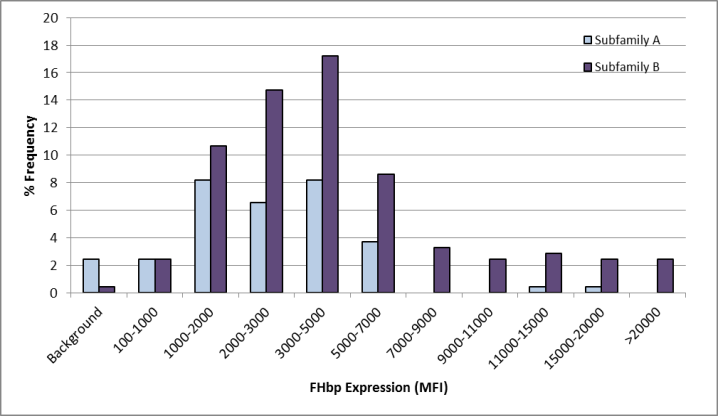

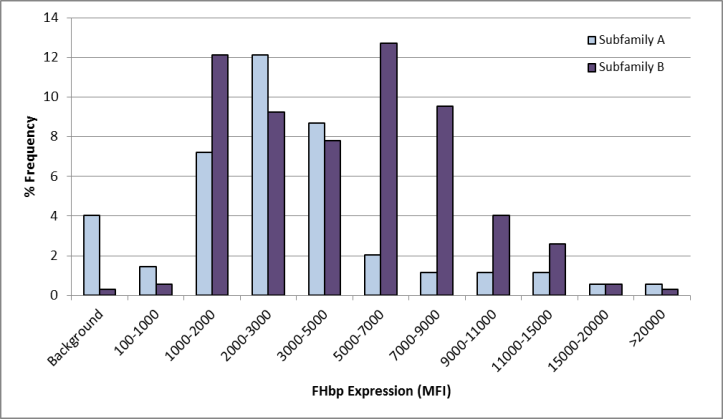

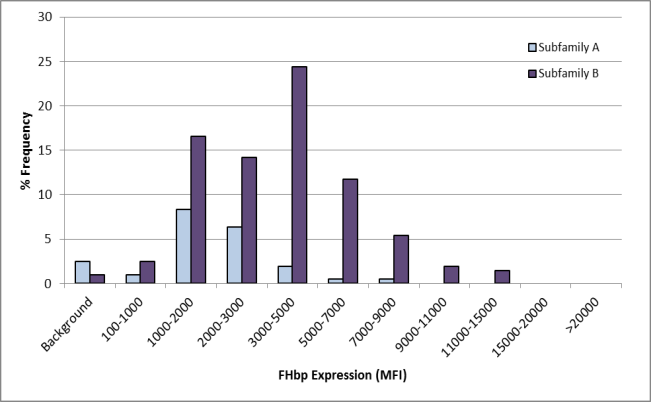

Supplement: FIG S6 [file mbo001183767sf6.docx]
