# Supplementary material for: Predicting the Susceptibility of Meningococcal Serogroup B Isolates to Bactericidal Antibodies Elicited by Bivalent rLP2086, a Novel Prophylactic Vaccine
Source: mBio. 2018 Mar 13;9(2):e00036-18. doi: 10.1128/mBio.00036-18 (PMC5850321; doi:10.1128/mBio.00036-18)
Supplement: TABLE S4 [file mbo001183767st4.docx]

**Supplemental Table S4. fHBP Expression Medians in Prevalent Variants for the NmB invasive isolate set**

| fHbp Variant | Prevalence  in the Strain  Pool (%) | fHbp Median Surface  Expression Levels  (MFI), (25^th^, 75^th^ Percentile) |
| --- | --- | --- |
| B24 | 23 | 7,518 (5583, 10229) |
| B16 | 11 | 1,966 (1629, 2508) |
| A22 | 10 | 2,519 (1769, 3425) |
| B03 | 9 | 2,907 (1815, 3948) |
| B44 | 8 | 14,378 (11047, 18848) |
| B09 | 6 | 2,058 (1641, 2608) |
| A12 | 3 | 2,132 (1232, 2997) |
| A19 | 3 | 2,278 (1140, 2901) |
| A06* | 2 | 3,008 (2053, 3522) |
| A05* | 2 | 1,827 (594, 4103) |
| A07* | 2 | 1,100 (429, 1660) |

* These variants have equal prevalence
